# Family History of Substance Use and Stressful Life Events Impact Adolescent Maturation of Cerebral White Matter

**DOI:** 10.1111/adb.70089

**Published:** 2025-10-19

**Authors:** Yizhou Ma, Ashley Acheson, Corneliu Bolbocean, Mustafa N. Mithaiwala, Si Gao, Neda Jahanshad, Paul M. Thompson, Bhim M. Adhikari, Xiaoming Du, A. Ankeeta, Alia Warner, Antonio F. Pagán, L. Elliot Hong, Peter Kochunov

**Affiliations:** ^1^ Department of Psychiatry and Behavioral Science University of Texas Health Science Center Houston Texas USA; ^2^ Department of Psychiatry University of Arkansas for Medical Sciences Little Rock Arkansas USA; ^3^ Division of Pharmaceutical Evaluation & Policy, Department of Pharmacy Practice University of Arkansas for Medical Sciences College of Pharmacy Little Rock Arkansas USA; ^4^ Imaging Genetics Center, Mark and Mary Stevens Neuroimaging and Informatics Institute, Keck School of Medicine of USC University of Southern California Marina del Rey California USA

**Keywords:** ABCD, family history, fractional anisotropy, longitudinal design, stressful life events, substance use disorders, white matter

## Abstract

Family history (FH) of substance use disorders (SUDs) and stressful life events (SLEs) are known risk factors for SUDs in adolescents and young adults. Cross‐sectional studies suggest that FH and SLEs affect adolescent white matter (WM) development and form abnormal WM patterns. Here, we examined the effects of FH, SLEs and their interaction on WM integrity in youths in the Adolescent Cognitive Brain Development (ABCD) study at baseline and 2‐ and 4‐year follow‐ups. ABCD youths (*N* = 8939, age ± SD = 9.9 ± 0.6 years, 4302 female) completed baseline diffusion tensor imaging, of which 5661 repeated the scan at 2‐year follow‐up (age ± SD = 12.0 ± 0.7 years, 2634 female) and 2177 at 4‐year follow‐up (age ± SD = 14.1 ± 0.7 years, 1007 female). FH was measured as the weighted sum of biological parents and grandparents with alcohol and/or drug problems. SLEs were measured with parental report of life events. WM integrity was measured with fractional anisotropy (FA) of 23 WM tracts. Linear mixed effect models were used to examine the effects of FH, SLEs and their interaction on FA at baseline and longitudinally, modelling family and study site as random intercepts and correcting for multiple comparisons with false discovery rate (FDR) *q* = 0.05. At baseline, there were no significant effects of FH, SLEs and their interaction on FA after multiple comparison correction when controlling for race, family income and parental education. From baseline to 4‐year follow‐up, FH significantly negatively interacted with newly occurred SLEs on FA in 19 out of 23 tracts, so that FA at 4‐year was lower in youths with both FH and newly occurred SLEs when controlling for baseline FA (*β*_interaction_ = −0.049 − −0.018, *p*_FDR_ = 6.2 × 10^−5^ − 4.7 × 10^−2^). These negative interactions were not significant with shorter time spans (baseline to 2‐year follow‐up and 2‐ to 4‐year follow‐up). In conclusion, we replicated findings from cross‐sectional cohorts of the effects of FH and SLEs on lower WM integrity in youths. The study utilized Big Data longitudinal design to show that FH‐by‐SLE interaction, rather than their independent effects was responsible for developmental WM changes associated with FH of SUDs and life stressors.

## Graphical abstract

In a large sample of adolescents (*N* = 2177, baseline age = 9.9 years), family history of substance use disorders and stressful life events had synergistic effects on widespread lower white matter integrity 4 years later.

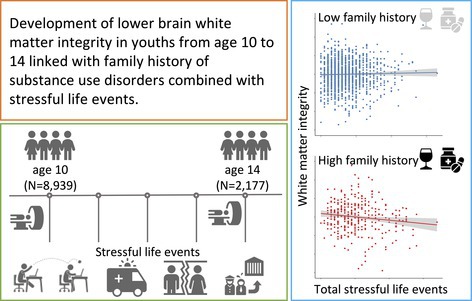

## 1 Introduction

Adolescence is an important developmental period associated with rapid myelination of associative cerebral white matter (WM), emergence of higher cognitive function and a heightened risk for developing substance use disorders (SUDs) [1, 2, 3, 4, 5]. Familial and environmental risks for SUDs are hypothesized to alter the brain's developmental trajectory, especially that of the cerebral WM tracts that interconnect frontal, limbic and parietal associative areas [5, 6] that are crucial for executive functioning and inhibition control [7, 8, 9]. Family history (FH) of SUDs and stressful life events (SLEs) are among the most replicable risk factors for SUDs in adolescents [10, 11, 12, 13, 14, 15]. Both risk factors are associated with altered WM microstructure even in adults who have never developed SUDs [16, 17, 18, 19, 20]. Most findings to date on SUD‐related WM changes were reported in cross‐sectional adult cohorts and included subjects with SUDs. Here, we analysed data from a longitudinal cohort of participants in the Adolescent Brain and Cognitive Development (ABCD) study starting from prepubescence.

FH‐SUDs are a chief risk factor for SUDs. Adolescents with FH‐SUDs are at risk to initiate substance use earlier, transition to heavy use and develop SUDs [10, 11, 21]. Importantly, FH may alter the normal development of brain circuits involved in executive control and reward processing [22, 23, 24]. Altered WM microstructure is consistently reported in frontocortical and frontostriatal tracts in adolescents with FH‐SUDs, including lower FA in the anterior and superior corona radiata, superior frontal‐occipital fasciculus, superior longitudinal fasciculus and anterior limb of the internal capsule [19, 25, 26]. These WM alterations likely reflect delayed or impaired axonal myelination based on combined radial diffusivity and magnetic resonance spectroscopy analyses [19, 27]. While some of these alterations may persist into adulthood, others may be transient and create a sensitive period when adolescents with FH‐SUDs are at risk for problematic substance use [19, 28, 29].

SLEs is a primary environmental risk factor for SUDs and many other psychopathologies that involve executive control and emotional regulation [12, 14, 30]. SLEs are a normal part of life and range in intensity and severity (e.g., deaths in the family, parental divorce, financial struggles and transition to a new school) [31, 32] and have a larger and longer lasting impact than SLEs during adulthood [33, 34, 35, 36, 37]. SLEs are hypothesized to contribute to risks of substance use in adolescents by interfering with the reward and emotion regulation circuits [38, 39, 40, 41, 42, 43, 44]. Exposure to SLEs is associated with lower FA in WM tracts including the corpus callosum, corona radiata, superior and inferior longitudinal fasciculus, uncinate fasciculus and the cingulum‐hippocampal portion [16, 17, 45, 46], many of which are important indicators associated with SUDs [47, 48, 49]. In one study, childhood/adolescent maltreatment was associated with lower FA in the hippocampal cingulum in adolescents around age 16, which predicted the development of SUDs at follow‐up ~3.5 years later [46].

Although FH‐SUDs and SLEs have separately been associated with WM alterations in cross‐sectional adolescent and adult cohorts, to our knowledge no study has evaluated their joint or interactive effects longitudinally. Longitudinal analysis of independent and interactive effects of FH‐SUDs and SLEs is key to identifying vulnerability biomarkers for early interventions aimed at disrupting pathogenic processes that lead to SUDs. In this study, we examined the effects of FH and SLEs on WM integrity in a large community‐based cohort of adolescents followed at baseline (age 9–10) and 2‐ and 4‐year follow‐ups. Based on previous findings, we hypothesize that both FH and SLEs are associated with lower WM FA in tracts including the corona radiata, corpus callosum, superior and inferior longitudinal fasciculus and cingulum at baseline. We further hypothesize that FH and SLEs will have significant interaction so that adolescents with both risk factors will have even more altered WM FA. Lastly, we predict that WM FA on a follow‐up visit will be explained by FH, new SLEs since a previous visit and their interaction, supporting longitudinal effects of FH and SLEs on WM development. We will test this hypothesis by comparing FA across 2‐ (baseline vs. 2‐year follow‐up and 2‐ vs. 4‐year follow‐up) and 4‐year (baseline vs. 4‐year follow‐up) intervals.

## 2 Methods

### 2.1 Participants

The Adolescent Cognitive Brain Development (ABCD) is a large prospective population‐based cohort study that followed risk factors, mental health and brain development of children across the United States starting from ages 9 to 10. Details of the ABCD study can be found in previous publications [50]. For this study, we used the ABCD data released to date (ABCD Release 5.1, November 2023). Child participants completed diffusion tensor imaging at baseline and 2‐ and 4‐year follow‐ups. Participants and their caregivers also completed demographic and clinical questionnaires at baseline and 1‐ to 4‐year follow‐ups.

### 2.2 FH of Alcohol and Drug Problems

FH of alcohol and drug problems (not including tobacco) was measured with the Family History Assessment Module Screener [51] completed by parents at baseline. Parents indicated for each blood relative of the participant if the relative had ever experienced problems due to alcohol and due to drugs, including ‘marital separation or divorce’, ‘laid off or fired from work’, ‘arrests or DUIs’, ‘alcohol/drugs harmed their health’, ‘in an alcohol/drug treatment program’, ‘suspended or expelled from school 2 or more times’ and ‘isolated self from family, caused arguments or were drunk/high a lot’. We quantified FH with weighted FH density, which is the number of biological parents (weighted 1) and grandparents (weighted 0.5) with any alcohol or drug problems [19, 52, 53]. Weighted FH density ranged from 0 (no biological parents or grandparents with alcohol or drug problems) to 4 (all biological parents and grandparents with alcohol or drug problems).

### 2.3 SLEs

SLEs were measured with the Adverse Life Events Scale [54, 55] completed by parents at annual follow‐ups. Twenty‐five SLEs were surveyed, including deaths and serious injury in family, witnessing crime or accident, losing a close friend, close friend seriously sick/injured, negative changes in parents' financial situation and having a new sibling. For each SLE, parents indicated whether the event was ever experienced by the participant, whether it was experienced in the past year, whether it was a good or bad experience and how much the experience affected the participant. As this questionnaire was not administered at baseline, we estimated baseline SLEs with data at 1‐year follow‐up: Baseline SLEs were the total number of SLEs reported as ever experienced at 1‐year follow‐up minus the total number of SLEs reported as experienced in the past year at the same visit. New SLEs between visits were quantified by adding the total number of SLEs that happened in the past year at relevant follow‐ups. For example, new SLEs between baseline and 2‐year follow‐up were the sum of the total number of SLEs in the past year at 1‐ and 2‐year follow‐ups. Following previous SLEs research in this age range, we used SLEs reported by parents because they are typically more accurate than reports by children in this age range, particularly regarding the timing of events [55, 56, 57]. We chose the total number of SLEs without accounting for how they were subjectively experienced (i.e., valence and intensity) as the former is a closer reflection of objectively experienced total stress (see [58] for a detailed discussion on considerations behind objective vs. perceived stress).

### 2.4 Neuroimaging Acquisition, Processing and Fractional Anisotropy (FA)

**FIGURE 1:**
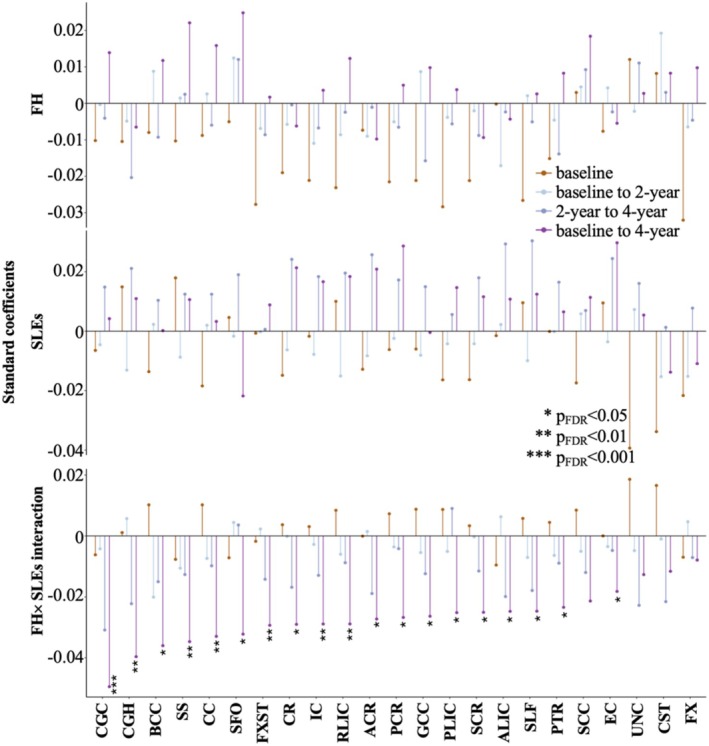
Effects of FH, SLEs and their interaction on WM FA. ACR: anterior corona radiata; ALIC: anterior limb of internal capsule; BCC: body corpus callosum; CC: corpus callosum; CGC: cingulum; CGH: cingulum (hippocampal portion); CR: corona radiata; CST: corticospinal tract; EC: external capsule; FDR: false discovery rate; FH: family history; FX: fornix; FXST: fornix–stria terminalis; GCC: genu of corpus callosum; IC: internal capsule; PCR: posterior corona radiata; PLIC: posterior limb of internal capsule; PTR: posterior thalamic radiation; RLIC: retrolenticular limb of internal capsule; SCC: splenium of corpus callosum; SCR: superior corona radiata; SFO: superior fronto‐occipital fasciculus; SLEs: stressful life events; SLF: superior longitudinal fasciculus; SS: sagittal striatum; UNC: uncinate.

The ABCD neuroimaging protocol and quality control and assurance are described elsewhere [59, 60]. Briefly, participants across 21 ABCD sites completed T1‐weighted (1 × 1 × 1 mm^3^), T2‐weighted (1 × 1 × 1 mm^3^) and diffusion‐weighted (1.7 × 1.7 × 1.7 mm^3^ with 96 diffusion directions and multiple *b* values) magnetic resonance imaging at baseline and 2‐ and 4‐year follow‐ups using Siemens, Philips or GE scanners. We used the Enhancing NeuroImaging Genetics through Meta‐Analysis data processing protocols for structural T1‐weighted and diffusion imaging data that were optimized for multisite processing and extraction of tract‐wise average FA values [61]. This resulted in FA values in 23 WM tracts in the JHU WM atlas (Figure 1) [62, 63]. We then used a mega‐analysis function, *polyclass*, in the software package SOLAR‐Eclipse (https://solar‐eclipse‐genetics.org/) to remove site scanner specific variances in average and distribution of FA values [64, 65]. Data normalization was performed in two steps: First, age, sex and intracranial volume covariates were regressed out of FA values per site; second, site‐wise inverse Gaussian normalization was performed to ensure normality. FA values across sites were then combined into a single mega‐analytical cohort and outliers that were more than six mean absolute deviations from the median of each WM tract on each visit were removed.

### 2.5 Substance Use Behaviour

The ABCD's substance use assessment is described elsewhere [66]. Briefly, participants completed a substance use interview at baseline to report their lifetime use of major drug categories. They also reported their drug use in the past 6 months (at baseline) and since the last visit (at annual follow‐ups) and completed a web‐based timeline follow‐back interview for each major drug category endorsed as used [66, 67]. They additionally completed mid‐year substance use phone interviews at 6, 18, 30 and 42 months about their substance use in the past 6 months. Following a previous study [68], we characterized SU behaviour as (1) SU experimentation, defined as a lifetime history of alcohol sipping or nicotine or cannabis puffing or tasting, and (2) SU initiation, defined as a lifetime history of at least 1 standard alcohol drink, nicotine or cannabis use more than puffing or tasting or trying or using any other substance. SU behaviour at 2‐ and 4‐year follow‐ups included all available on‐site and phone interviews from baseline to the relevant follow‐up.

### 2.6 Statistics

To examine baseline effects of FH, SLEs and their interaction on WM FA, we regressed FA of each WM tract onto weighted FH density, baseline total SLEs and their interaction in linear mixed effect models, modelling random intercepts for families nested within sites. Main effects of FH and SLEs and interaction effects on the 23 WM tracts were each corrected for multiple comparisons using false discovery rate *q* = 0.05.

To examine longitudinal effects of FH, new SLEs from baseline to 2‐year follow‐up and their interaction on FA from baseline to 2‐year follow‐up, we regressed 2‐year FA of each WM tract onto weighted FH density, total SLEs from baseline to 2‐year follow‐up and their interaction in linear mixed effect models, controlling for baseline FA and modelling random intercepts for families nested within sites. Multiple comparison correction was the same as the baseline analysis. The same analysis was repeated for FA from 2‐ to 4‐year follow‐ups and from baseline to 4‐year follow‐up.

All models controlled for covariates including age, sex and sociodemographic variables (i.e., race, parental education and family income). We classified race groups including White (64.1% at baseline), Black (15.5%) and Asian (2.1%, including Chinese, Vietnamese, Asian Indian, etc.). Participants who were considered by their parents to be multiracial or of races uncovered by the ABCD survey are grouped in an ‘Other’ group (16.8%), which also included American Indian and Pacific Islander which together accounted for less than 1% of the sample. Parental education was classified as ‘below high school’, ‘high school/GED’, ‘some college’, ‘college’ and ‘postgraduate’ based on the parent with the higher level of education. Family income was classified as ‘low’ (below $50 000), ‘medium’ ($50 000–$99 999) and ‘high’ ($100 000 and greater) based on total combined income over the past 12 months.

## 3 Results

### 3.1 Sample Characteristics

At baseline, *N* = 8939 (age ± SD = 9.9 ± 0.6 years, 4302 female) participants had usable diffusion imaging data (Table 1). Among these participants, *N* = 5661 (age ± SD = 12.0 ± 0.7 years, 2634 female) also had 2‐year and *N* = 2177 (age ± SD = 14.1 ± 0.7 years, 1007 female) had 4‐year usable diffusion imaging data (only ~50% imaging data at 4 years have been released to date).

**TABLE 1:** Characteristics of participants with diffusion imaging data at baseline and 2‐ and 4‐year follow‐ups.

	Baseline	2‐year follow‐up	4‐year follow‐up
*N*	8939	5661	2177
Age (years)			
Mean ± SD	9.9 ± 0.6	12.0 ± 0.7	14.1 ± 0.7
Sex (%)			
M	51.8	53.4	53.7
F	48.1	46.5	46.3
Race (%)			
White	64.1	67.3	65.6
Black	15.4	13.3	13.6
Asian	2.1	1.7	1.8
Other	16.8	16.4	17.2
Parental education (%)			
Below HS	3.8	3.9	3.7
HS/GED	8.1	8.4	9.0
Some college	29.8	32.8	32.7
College	20.0	22.2	22.7
Postgraduate	30.5	32.3	31.6
Income (%)			
Low	26.7	25.4	27.4
Medium	26.1	28.2	27.7
High	39.1	38.9	37.9
FH of substance use problems (%)			
Alcohol only	19.3	19.2	18.7
Other drug only	4.4	4.3	4.3
Alcohol and other drugs	9.0	9.6	9.5
Weighted FH density			
Mean ± SD	0.36 ± 0.63	0.36 ± 0.63	0.37 ± 0.64
Baseline total SLEs			
Mean ± SD	1.5 ± 2.0	1.5 ± 2.0	1.5 ± 2.0
Substance experimentation (%)			
Sipped alcohol	22.3	30.3	37.7
Puffed nicotine	0.7	1.7	5.6
Puffed/tasted cannabis	< 0.1	0.4	3.0
Any of the above	22.6	31.0	39.9
Substance initiation (%)			
Alcohol use (≥ 1 drink)	0.2	0.9	3.3
Nicotine use	0.5	1.3	4.5
Cannabis use	< 0.1	1.1	3.1
Other drug use	0.1	0.9	2.4
Any of the above	0.8	3.7	9.6

*Note:* Race: Other included other, mixed races, American Indian and Pacific Islander. Income: Low: less than $50 000; Medium: $50 000–$99 999; High: $100 000 or greater.

Abbreviations: FH: family history; HS: high school; SD: standard deviation; SLEs: stressful life events.

Weighted FH density in the baseline sample averaged around 0.36 with a standard deviation of 0.63 (range = 0–4). In a linear mixed effect model controlling for the random effects of family and site, weighted FH density was significantly higher in participants in White (mean = 0.42) or Other (mean = 0.44) than Black (mean = 0.28) or Asian (mean = 0.24) groups after adjusting for parental education and family income, *p* = 1.6 × 10^−8^. Weighted FH density was highest in participants with parental education of ‘high school/GED’ (mean = 0.39) and ‘some college’ (mean = 0.45), which was significantly higher than ‘below high school’ (mean = 0.35) and ‘college’ (mean = 0.30), and lowest in ‘postgraduate’ (mean = 0.24) after adjusting for race and family income, *p* < 2.2 × 10^−16^. Weighted FH density was significantly lower as family income increased from ‘low’ (mean = 0.43) to ‘medium’ (mean = 0.34) to ‘high’ (mean = 0.26) after adjusting for race and parental education, *p* = 1.4 × 10^−10^.

Total number of SLEs in the baseline sample averaged around 1.5 with a standard deviation of 2.0 (range = 0–15). In a linear mixed effect model controlling for the random effects of family and site, total number of SLEs at baseline was significantly higher in participants in White (mean = 1.1) or Other (mean = 1.0) than Black (mean = 0.6) or Asian (mean = 0.5) race groups after adjusting for age, sex, parental education and family income, *p* = 1.3 × 10^−9^. Total number of SLEs at baseline was significantly higher in participants with parental ‘postgraduate’ (mean = 1.1), ‘college’ (mean = 1.0) or ‘some college’ (mean = 1.0) than ‘high school/GED’ (mean = 0.7) or ‘below high school’ (mean = 0.3) education after adjusting for age, sex, race and family income, *p* = 1.2 × 10^−7^. Total number of SLEs at baseline was significantly lower as family income increased from ‘low’ (mean = 1.0) to ‘medium’ (mean = 0.9) to ‘high’ (mean = 0.5) after adjusting for age, sex, race and parental education, *p* = 4.6 × 10^−11^.

Generalized linear mixed effect models did not suggest that weighted FH density or baseline total number of SLEs was associated with diffusion data availability at follow‐up visits (all *p*s > 0.22). From baseline to 2‐year follow‐up, participants with repeated diffusion imaging data had 5.0 ± 3.7 new SLEs (range = 0–29). From 2‐ to 4‐year follow‐ups, participants had 3.0 ± 2.6 new SLEs (range = 0–22).

At baseline, 22.6% of the participants endorsed lifetime SU experimentation, mostly in the form of alcohol sipping (22.3%). Only 0.8% of the participants endorsed lifetime SU initiation, including 0.5% of the participants with nicotine use more than puffing. At the 2‐year follow‐up, lifetime SU experimentation increased to 31.0% and initiation increased to 3.7%. At the 4‐year follow‐up, 39.9% of the participants endorsed SU experimentation and 9.6% endorsed SU initiation. These estimates are similar to the previous characterization of the same cohort, supporting a lower rate of substance use in the currently released ABCD data [68]. Generalized linear mixed effect models did not suggest that baseline SU experimentation or initiation was associated with diffusion data availability at follow‐up visits (all *p*s > 0.10).

### 3.2 Baseline Effects of FH and SLEs on WM FA

At baseline, there were no significant effects of FH, SLEs and their interaction on any tract after multiple comparison correction (Figure 1). Uncorrected results suggested that weighted FH density was associated with lower FA in the fornix (FX, *β* = −0.032, *p*_uncorrected_ = 0.009), fornix–stria terminalis (FXST, *β* = −0.028, *p*_uncorrected_ = 0.024) and posterior limb internal capsule (PLIC, *β* = −0.028, *p*_uncorrected_ = 0.041), and more SLEs were associated with lower FA in the uncinate fasciculus (UNC, *β* = −0.039, *p*_uncorrected_ = 0.013) and corticospinal tract (CST, *β* = −0.034, *p*_uncorrected_ = 0.034).

### 3.3 Longitudinal Effects of FH and SLEs on WM FA

**FIGURE 2:**
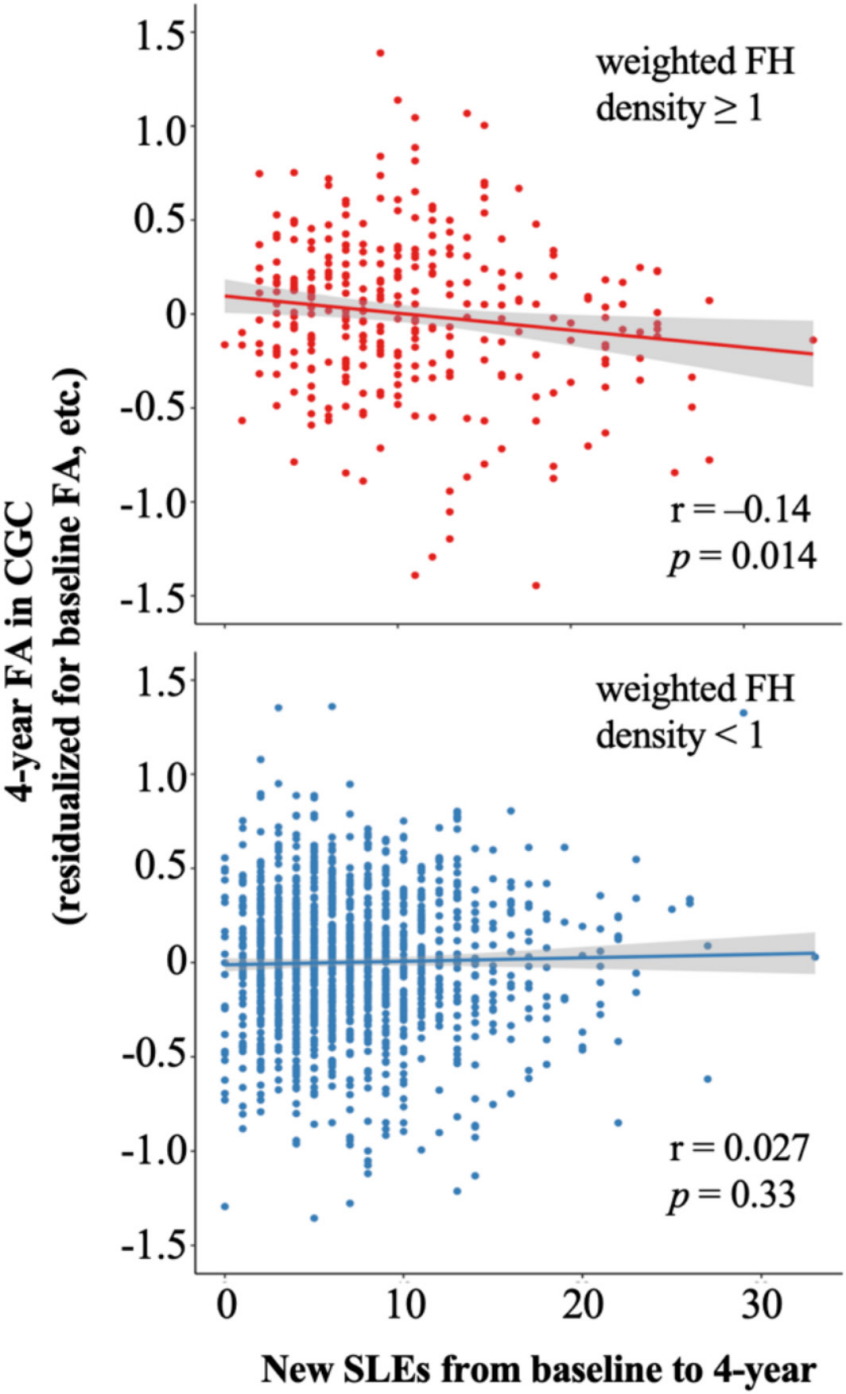
Negative interaction between FH and new SLEs from baseline to 4‐year follow‐up on FA. CGC: cingulum; FA: fractional anisotropy; FH: family history; SLEs: stressful life events. FA values in the *y* axis are residualized for baseline FA and other covariates.

FH, new SLEs and their interaction had no significant effect on FA from baseline to 2‐year follow‐up and from 2‐ to 4‐year follow‐ups after multiple comparison correction (Figure 1). From baseline to 4‐year follow‐up, a significant negative interaction between weighted FH density and new SLEs was found on 19 out of 23 WM tracts, including the cingulum (CGC) and its hippocampal portion (CGH), internal capsule (retrolenticular [RLIC], anterior [ALIC] and PLIC and whole), corpus callosum (body [BCC] and genu [GCC] aspects and whole), corona radiata (anterior [ACR], superior [SCR], posterior [PCR] and whole), sagittal striatum (SS), FXST, superior longitudinal fasciculus (SLF), posterior thalamic radiation (PTR), superior fronto‐occipital fasciculus (SFO) and external capsule (EC) (*β*_interaction_ = −0.049 − −0.018, *p*_FDR_ = 6.2 × 10^−5^ − 4.7 × 10^−2^). In these tracts, participants with higher weighted FH density and more SLEs from baseline to 4‐year follow‐up had lower FA at 4‐year follow‐up when controlling for baseline FA. The strongest interaction effects were found in the CGC (*β*_interaction_ = −0.049, *p*_FDR_ = 6.2 × 10^−5^) and CGH (*β*_interaction_ = −0.039, *p*_FDR_ = 1.4 × 10^−3^). Figure 2 illustrates the negative interaction in CGC. In participants with weighted FH density equal to or larger than 1 (i.e., at least one biological parent or at least two biological grandparents with alcohol or drug problems), more new SLEs from baseline to 4‐year follow‐up were associated with significantly lower 4‐year FA in the CGC after controlling for baseline FA and other covariates (*r* = −0.14, *p* = 0.014). In participants with weighted FH density less than 1 (i.e., alcohol or drug problems not present or limited to one biological grandparent), new SLEs were not associated with 4‐year FA.

Although the models investigating changes from baseline to 2‐year follow‐up and from 2‐ to 4‐year follow‐ups were not significant after multiple comparisons, some of the 19 WM tracts that showed significant interaction from baseline to 4‐year follow‐up also had interaction effects in the negative direction during these shorter intervals. From baseline to 2‐year follow‐up, 14 out of the 19 WM tracts also had interaction effects in the negative direction, including significant interaction before multiple comparison correction in the BCC (*β*_interaction_ = −0.020, *p*_uncorrected_ = 2.6 × 10^−2^). From 2‐ to 4‐year follow‐up, 17 out of the WM tracts also had interaction effects in the negative direction, including significant interaction before multiple comparison correction in the CGC (*β*_interaction_ = −0.030, *p*_uncorrected_ = 8.9 × 10^−3^).

### 3.4 WM FA and SU Behaviour

Lastly, we conducted exploratory analyses to see if lower FA in the 19 WM tracts showing interactive effects between FH‐SUDs and SLEs was related to SU behaviour. Linear mixed effect models showed that compared to participants with no changes in SU, participants who first experimented with SU between baseline and 4‐year follow‐up had lower FA at 4‐year follow‐up when controlling for baseline FA in the CGH, which approached significance (*B =* −0.051, *p*_uncorrected_ = 5.2 × 10^−2^). No significant effects were found for SU initiation. Controlling for SU initiation or experimentation did not change the effects of FH‐SUD, SLEs and their interactions on WM FA.

## 4 Discussion

We examined the effects of key familial and environmental risk factors for problematic substance use (FH‐SUDS and SLEs) on longitudinal WM integrity of the ABCD cohort using baseline and 2‐ and 4‐year follow‐ups. We found no significant effects of FH‐SUDs and SLEs on baseline FA values. Instead, the changes in cerebral WM from 10 to 14 years were significantly affected by the FH‐SUDs × SLEs interaction. New cumulative SLEs exposure from age 10 to 14 years was associated with lower rates of maturation in 19 out of 23 WM tracts in adolescents with FH of SUDs. These findings highlight the critical influence of environmental stressors on brain development in adolescents with FH of SUDs. They also highlight the importance of considering the interaction between risk factors when examining their effects on WM development and substance use. Adolescents with compounding risks for substance use may require research and clinical priority.

We observed no significant baseline effects of FH on WM FA that were reported in other samples of adolescents with FH of SUDs [19, 25, 26, 27, 69]. One key difference between the cohorts is that the ABCD sample is younger at baseline (average age around 10), as compared to 12–16 years old in other studies. FH‐SUDs are hypothesized to alter the maturation of WM tracts that are specifically developing during adolescence [19]. Therefore, the lack of baseline FH‐SUDs effects may suggest more nuanced and age‐specific ways in which FH‐SUDs affect cerebral maturation, including through its interaction with SLEs [68].

We demonstrated that FH‐SUDs by SLEs interaction was associated with delayed or altered maturation of cerebral WM. These effects were significant for 19 out of 23 WM tracts, replicating findings of lower FA values in adolescents with FH‐SUDs (e.g., CC, ACR and SLF) reported in FH‐enriched cohorts [19, 25, 26, 27], with the strongest effects observed in the cingulum. The cingulum is an important WM tract that interconnects the prefrontal cortex, hippocampus and amygdala [70, 71] and mediates stress reactivity [72]. Altered cingulum FA is reported in childhood maltreatment, combat exposure and post‐traumatic stress disorder [73, 74, 75]. Moreover, alterations in the cingulum were reported in meta‐analyses of disordered methamphetamine, alcohol and polysubstance use [29, 47, 49]. Thus, the interaction between new SLEs and FH on reduced FA from baseline to 4‐year follow‐up may reflect heightened vulnerability of adolescents with FH of SUD to SLEs in their stress regulation circuits, which sets the stage for maladaptive substance use. Although we did not find the same significant interaction at shorter follow‐up intervals (i.e., from baseline to 2‐year follow‐up and from 2‐ to 4‐year follow‐up), the interaction effects were in the same negative direction for most of the WM tracts and were nominally significant from 2‐ to 4‐year follow‐ups in the CGC. WM vulnerability to SLEs in individuals with FH of SUD may be age sensitive or may need a longer time interval and a higher number of accumulated SLEs to be observed.

We did not find a significant association between FA of the 19 WM tracts showing FH‐by‐SLE interaction and SU behaviour, except for an association between lower FA in the CGH at 4‐year follow‐up (controlling for baseline) and the first SU experiment between baseline and 4‐year follow‐up that approached significance in exploratory analyses. Given the lower rate of SU in the ABCD by 4‐year follow‐up, we may not have had enough power to detect the effect of WM FA on SU behaviour. Further analysis in future data releases is needed to determine if FH‐SUDs, SLEs and the WM deficits identified in this study will be associated with SU and other undesired outcomes as ABCD participants age.

This study has strengths and limitations. The ABCD dataset is an unprecedented resource for longitudinally examining influences such as FH‐SUDs and SLEs on adolescent brain development. However, ABCD participants were more likely to be of higher socioeconomic status, have married and working parents and live in urban areas than their US birth cohort [76], which may limit how well findings generalize. Like commonly done in other studies in children with FH‐SUDs, FH was determined through parent report. However, it is possible that some respondents may not have been aware of all problem substance use in family members. Likewise, relying on parent report of children's SLEs increased the accuracy of responding; however, it is possible that some events may have gone unreported due to parents not being aware of them or having concerns about potential repercussions of reporting them. Lastly, SLEs represent only one aspect of stress during adolescence. Future studies should explore the impact of more broadly defined adversity, including family conflicts, neighbourhood crime and environmental pollution, on WM development [77, 78, 79].

## 5 Conclusion

FH of SUDs and SLEs are risk factors of problematic substance use in adolescents. Adolescents with dual risks are particularly vulnerable to altered development in a wide array of WM tracts over time, which may set the stage for their substance use and related problems. Research and clinical practice should pay more attention to adolescents with compounding risks of altered brain development and substance use.

## Author Contributions

**Yizhou Ma:** conceptualization, methodology, formal analysis, writing – original draft, visualization. **Ashley Acheson:** conceptualization, methodology, writing – review and editing, funding acquisition. **Corneliu Bolbocean:** conceptualization, writing – review and editing. **Mustafa N. Mithaiwala:** conceptualization, writing – review and editing. **Si Gao:** data curation, writing – review and editing. **Neda Jahanshad:** writing – review and editing. **Paul M. Thompson:** writing – review and editing, funding acquisition. **Bhim M. Adhikari:** writing – review and editing. **Xiaoming Du:** writing – review and editing. **A. Ankeeta:** writing – review and editing. **Alia Warner:** writing – review and editing. **Antonio F. Pagán:** writing – review and editing. **L. Elliot Hong:** writing – review and editing, funding acquisition. **Peter Kochunov:** conceptualization, methodology, writing – review and editing, funding acquisition.

## Conflicts of Interest

The authors declare no conflicts of interest.

## Data Availability

The data that support the findings of this study are openly available in ABCD Study's Data Release 5.1 at https://doi.org/10.15154/z563‐zd24.
